# Dietary Intake and Body Mass Index Influence the Risk of Islet Autoimmunity in Genetically At-Risk Children: A Mediation Analysis Using the TEDDY Cohort

**DOI:** 10.1155/2023/3945064

**Published:** 2023-02-17

**Authors:** Carin Andrén Aronsson, Roy Tamura, Kendra Vehik, Ulla Uusitalo, Jimin Yang, Michael J. Haller, Jorma Toppari, William Hagopian, Richard A. McIndoe, Marian J. Rewers, Anette-G. Ziegler, Beena Akolkar, Jeffrey P. Krischer, Jill M. Norris, Suvi M. Virtanen, Helena Elding Larsson

**Affiliations:** ^1^Department of Clinical Sciences, Lund University, Malmo, Sweden; ^2^Health Informatics Institute, Department of Pediatrics, Morsani College of Medicine, University of South Florida, Tampa, FL, USA; ^3^University of Florida Diabetes Institute, Gainesville, FL, USA; ^4^Department of Pediatrics, Turku University Hospital, Turku, Finland; ^5^Institute of Biomedicine, Research Centre for Integrative Physiology and Pharmacology, and Centre for Population Health Research, University of Turku, Turku, Finland; ^6^Pacific Northwest Research Institute, Seattle, WA, USA; ^7^Center for Biotechnology and Genomic Medicine, Medical College of Georgia, Augusta University, Augusta, GA, USA; ^8^Barbara Davis Center for Childhood Diabetes, University of Colorado, Aurora, CO, USA; ^9^Institute of Diabetes Research, Helmholtz Zentrum München and Klinikum rechts der Isar, Technische Universität München, Forschergruppe Diabetes e.V, Neuherberg, Germany; ^10^National Institute of Diabetes and Digestive and Kidney Diseases, Bethesda, MD, USA; ^11^Department of Epidemiology, University of Colorado Denver, Colorado School of Public Health, Aurora, CO, USA; ^12^Finnish Institute for Health and Welfare, Department of Public Health and Welfare, Helsinki, Finland; ^13^Faculty of Social Sciences, Unit of Health Sciences, Tampere University, Tampere, Finland; ^14^Center for Child Health Research, Tampere University and University Hospital, Tampere, Finland and Research, Development, and Innovation Center, Tampere University Hospital, Tampere, Finland; ^15^Department of Pediatrics, Skane University Hospital, Malmo, Lund, Sweden

## Abstract

**Results:**

We found an indirect effect of total energy intake (estimates: indirect effect 0.13 [0.05, 0.21]) and energy from protein (estimates: indirect effect 0.06 [0.02, 0.11]), fat (estimates: indirect effect 0.03 [0.01, 0.05]), and carbohydrates (estimates: indirect effect 0.02 [0.00, 0.04]) (kcal/day) on the development of IA. A direct effect was found for protein, expressed both as kcal/day (estimates: direct effect 1.09 [0.35, 1.56]) and energy percentage (estimates: direct effect 72.8 [3.0, 98.0]) and the development of GAD autoantibodies (GADA). In the sensitivity analysis, energy from protein (kcal/day) was associated with increased risk for GADA, hazard ratio 1.24 (95% CI: 1.09, 1.53), *p* = 0.042.

**Conclusions:**

This study confirms that higher total energy intake is associated with higher BMI, which leads to higher risk of the development of IA. A diet with larger proportion of energy from protein has a direct effect on the development of GADA.

## 1. Introduction

Type 1 diabetes is a chronic autoimmune disease characterized by destruction of the pancreatic islet beta-cells resulting in lifelong dependency on insulin replacement therapy. The clinical onset is preceded by an asymptomatic period, in which several autoantibodies to beta-cell antigens are detectable in serum, often termed islet autoimmunity. The development of multiple autoantibodies is highly predictive of type 1 diabetes [1]. Incidence of childhood type 1 diabetes has been trending upward with an estimated overall annual increase of 3% in many high- and low-income countries, suggesting a potential role for several environmental factors [2]. One environmental factor associated with the development of type 1 diabetes is rapid growth and obesity [3].

Weight gain, especially during early childhood (before the age of 2 years), has been suggested to predict the risk of islet autoimmunity in genetically predisposed children [4, 5], particularly in those children developing GAD65 autoantibodies (GADA) but not insulin autoantibodies (IAA) as their first appearing autoantibody [6].

It has been proposed that rapid childhood growth and weight gain may promote islet autoimmunity by creating an increased demand of insulin from the beta-cells. This would lead to a greater stress on the beta-cells and make them more susceptible to an autoimmune attack due to another triggering factor, such as enteroviral infections [7]. The accelerator hypothesis [8] proposes that excess weight gain leads to insulin resistance in early childhood and may initiate islet autoimmunity, eventually leading to type 1 diabetes.

Previous studies have focused on early growth and reported an association with both islet autoimmunity and progression to type 1 diabetes [4, 5]. In our first analysis of growth and risk for islet autoimmunity and type 1 diabetes in a multinational birth cohort on genetically at-risk children followed up to the age of 4 years, we reported a weak association between weight *z*-scores and risk for islet autoimmunity [9]. More recently, different growth phases during early childhood have been identified within the same cohort and analysis suggested an association between the early growth phase and the development of islet autoimmunity [6]. It is yet to be determined what aspect of growth, or factors influencing growth, maybe driving the association. As growth in children is directly related to dietary intake, exploration of the composition of the diet may lead to insights into the pathogenesis of islet autoimmunity.

Our aim was to estimate the effect of total energy and energy-yielding macronutrient intake on the development of islet autoimmunity in genetically at-risk children aged 2 to 8 years through body mass index (BMI), a prespecified mediator variable.

## 2. Methods

### 2.1. Study Population

The Environmental Determinants of Diabetes in the Young (TEDDY) is a prospective birth cohort consisting of 8,676 enrolled genetically at-risk children born between September 2004 and February 2010. Children were enrolled to the study before the age of 4.5 months and followed for 15 years to identify environmental triggers of type 1 diabetes [10]. Children carrying high-risk human leukocyte antigen (HLA) alleles for type 1 diabetes were enrolled at six centers: three in the USA (Colorado, Washington, and Georgia/Florida) and three in Europe (Finland, Germany, and Sweden). The following HLA-class II genotypes were eligibility criteria for enrollment to the study from the general population: DRB1 *∗* 04-DQA1 *∗* 03-DQB1 *∗* 03 : 02/DRB1 *∗* 03-DQA1 *∗* 05-DQB1 *∗* 02 : 01 (DR3/4), DRB1 *∗* 04-DQA1 *∗* 03-DQB1 *∗*03 : 02/DRB1 *∗* 04-DQA1 *∗* 03-QB1 *∗* 03 : 02 (DR4/4), DRB1 *∗* 04-DQA1 *∗* 03-DQB1 *∗* 03 : 02/DRB1 *∗* 08-DQA1 *∗* 04-DQB1 *∗* 04 : 02 (DR4/8), and DRB1 *∗* 03-DQA1 *∗* 05-DQB1 *∗* 02 : 01/DRB1 *∗* 03-DQA1 *∗* 05-DQB1 *∗* 02 : 01 (DR3/3). Infants with HLA-DR genotypes DRB1 *∗* 04-DQA1 *∗* 03-DQB1 *∗* 03 :0 2/DRB1 *∗* 04-DQA1 *∗* 03-DQB1 *∗* 02 : 02 (DR4/4b), DRB1 *∗* 04-DQA1 *∗* 03-DQB1 *∗* 03 : 02/DRB1 *∗* 01-DQA1 *∗* 01-DQB1 *∗* 05 : 01 (DR4/1), DRB1 *∗* 04-DQA1 *∗* 03-DQB1 *∗* 03 : 02/DRB1 *∗* 13-DQA1 *∗* 01-DQB1 *∗* 06 : 04 (DR4/13), DRB1 *∗* 04-DQA1 *∗* 03-DQB1 *∗* 03 : 02/DRB1 *∗* 09-DQA1 *∗* 03-DQB1 *∗* 03 : 03 (DR4/9), and DRB1 *∗* 03-DQA1 *∗* 05-DQB1 *∗* 02 : 01/DRB1 *∗* 09-DQA1 *∗* 03-DQB1 *∗* 03 : 03 (DR3/9) were included only if they had a first-degree relative (i.e., mother, father, or sibling) with type 1 diabetes. The HLA DR-DQ genotype abbreviations shown in parentheses will be used throughout this paper. Details of the study design, eligibility, and methods have been previously published [11].

**Figure 1:**
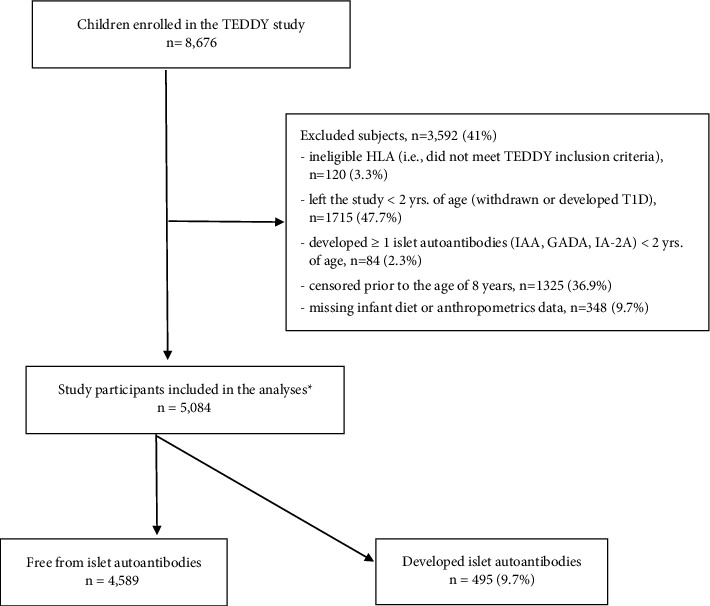
Flow chart describing the study population. ^*∗*^The outcome was islet autoantibody positive or negative by 8 years of age. No further observation of outcome was done after this time.

Subjects who developed islet autoantibodies or type 1 diabetes before the age of 2 years were excluded. A flowchart of the study populations in Figure 1 illustrates that a total of 5,084 children were prospectively followed from age 2 up to the age of 8 years for anthropometric measurements and food records with a median follow-up time of 6 years (IQR 5, 6). Written informed consent was obtained for all study participants from a parent or primary caretaker, separately, for genetic screening and participation in the prospective follow-up. The study was approved by local institutional review boards and is monitored by an external advisory board formed by the National Institutes of Health.

### 2.2. Anthropometric Variables and Growth Measures

Length and height were measured by trained study personnel at clinical visits every three months from the age of 3 months up to 4 years of age, and every 3 to 6 months after the age of 4 years. Length was measured as standing height to the nearest 0.1 cm. Body weight was measured in light clothing, using regularly calibrated digital scales. BMI (*z*-score) was calculated as weight in kilograms divided by height in meters squared and transformed to standard deviation scores using World Health Organization (WHO) reference values [12] (i.e., measures of relative weight adjusted for child age and sex) and was used as a continuous variable in our analyses.

### 2.3. Dietary Assessment

Data on food consumption were collected by 3-day food records, biannually from the age of 12 months. Parents were asked to keep a food record documenting all foods and beverages consumed by the child over a 3-day period (ideally two weekdays and one weekend day) within 10 days of the scheduled clinic visit. Detailed instructions on how to complete the food records were given to the parents, both verbal and written instructions. Additional guidance was given to the parents in the form of a food portion size booklet with photographs of foods and dishes containing four to five portion sizes, drawings, and shapes of other types of foods to enhance the accuracy in reporting the child's habitual food consumption. The dietary assessment method used in the study has been described previously [13]. When the child started attending daycare, separate food records and supporting material were provided for the daycare personnel. Completed food records were reviewed by a trained study nurse and family members were probed about missing information during face-to-face interview at the clinic visit. The food records were entered in country-specific food composition databases and each country analysed their food records separately. The four countries' food composition databases have been harmonized within the TEDDY study and values for energy and macronutrients (protein, fat, and carbohydrates) are comparable between all four databases [14]. Total mean energy intake from the reported days was calculated as mean intake, expressed as kilocalories (kcal). Energy from the energy-yielding macronutrients (protein, fat, and carbohydrates) was calculated as daily intake (in gram) and multiplied with the energy values for protein (4 kcal/g), fat (9 kcal/g), and carbohydrates (4 kcal/g). The proportion of energy-yielding macronutrients in the diet was calculated using energy from macronutrients divided by total energy intake, expressed as percentages (*E*%).

### 2.4. Study Outcomes

Venous blood samples were obtained every 3 months until the age of 4 years and biannually thereafter for the analysis of islet autoantibodies against insulin (IAA), glutamic acid decarboxylase (GADA), and insulinoma antigen-2 (IA-2A). In this study, islet autoimmunity was defined as persistent and confirmed positivity for ≥1 islet autoantibody in two or more consecutive blood draws, three months apart. Autoantibody titers have not been taken into account. Autoantibodies were measured in radiobinding assays as previously described [15].

### 2.5. Statistical Analyses

Mediation analysis for time-varying mediators and exposure was conducted by the method described by Lin et al. [16]. Mediation analyses does not imply causal relationships but quantifies the effect of the exposure variable on the outcome either directly (direct effect) or indirectly through the mediator (indirect effect). Mediation analysis for time-varying covariates, such as energy intake and BMI, has been described [17], and the possible relationship between reported energy intake and BMI with islet autoimmunity is depicted in the diagram shown in Supplementary Figure 1. This methodology requires that the event outcome to be known at all time points of the exposure period (i.e., no censoring). Thus, subjects who withdrew from the study between 2 and 8 years of age without reported autoantibodies (*n* = 1325) were excluded from this analysis in addition to subjects with ineligible HLA genotypes, missing infant diet or anthropometric data, or developed islet autoantibodies before the age of 2 years.

Three outcome events were examined: IAA, GADA, or any islet autoantibody (IAA, GADA, or IA-2A). For each outcome event, four different exposure variables were independently analysed: total energy and energy (calories) from protein, fat, and carbohydrates, respectively. For each outcome and exposure variable, BMI *z*-score was the mediator variable. Baseline covariates for all analyses were variables previously identified as risk factors associated with type 1 diabetes in the TEDDY study: country, first-degree relative with type 1 diabetes, and HLA genotype [18]. For IAA and GADA analyses, any other autoantibody detected was a time-varying binary confounder. A sensitivity analysis was conducted using a Cox proportional hazard model including all subjects who were autoantibody negative and with at least one submitted food record after the age of 2 years (*n* = 6,409). The analysis was stratified by country and included information about HLA-genotype and type 1 diabetes-FDR as baseline covariates and BMI *z*-score as time-varying covariate. The SAS macro, mgformula version 3, was used for all analyses.

## 3. Results

The cohort consists of 5,084 subjects who were islet autoantibody negative at the age of 2 years (Figure 1), as of July 31, 2020. At the last visit through the age 8 years, 495 (9.7%) were autoantibody positive, 319 (6.3%) with IAA, and 363 (7.1%) with GADA as the first appearing persistent and confirmed autoantibody. The median age of children at the time of seroconversion to islet autoimmunity in this study was 39 months (IQR = 36). The baseline characteristics for the study population are summarized in Table 1. Reported total energy intake and energy-yielding macronutrients increased gradually by age, while when expressed as percentages of energy, they remained relatively stable (Table 1).

The mediation analysis results in estimates of the total, direct, and indirect effects for each outcome and exposure combination (Table 2). The direct effect measures the association of the exposure on the outcome not going through the mediator (BMI *z*-score). The indirect effect measures the association of the exposure on the outcome going through the mediator.

There was no evidence of a direct effect of total energy intake or energy from protein, fat, or carbohydrates, respectively, on the development of islet autoimmunity, except for energy from protein (kcal/day) on the development of GADA positivity (estimates: direct effect 1.09 [0.35, 1.56]) (Table 2). In contrast, we found an indirect effect of total energy intake (estimates: indirect effect 0.13 [0.05, 0.21]) and energy from protein (estimates: indirect effect 0.06 [0.02, 0.11]), fat (estimates: indirect effect 0.03 [0.01, 0.05]), and carbohydrates (estimates: indirect effect 0.02 [0.00, 0.04]) on the development of islet autoimmunity (Table 2).

When repeating the analyses using energy percentage (energy from macronutrients divided by total energy intake) as exposure variables, the combination of energy from protein (%) and GADA positivity again showed a direct effect (estimates: direct effect 72.8 [3.0, 98.0]) (Table 3).

The analyses suggest that there is an indirect effect of total energy through BMI for the development of islet autoimmunity. To determine the effect size of BMI *z*-score on islet autoimmunity, a cross-tabulation was done with BMI *z*-score dichotomized at ≥1.5. Again, all subjects were autoantibody negative at the age of 2 years and the percentage of subjects with islet autoimmunity tends to be higher for the higher BMI subjects than for subjects with lower BMI *z*-scores, especially at age 3 (BMI *z*-score ≥1.5 3.4% vs. BMI *z*-score <1.5 2.0%) (Supplementary Table 1).

### 3.1. Sensitivity Analyses

A sensitivity analysis was conducted to determine whether the observed energy from protein (kcal/day) and GADA effect was due to the exclusion of subjects who withdrew from the study between the age of 2 and 8 years. The Cox regression analysis showed that energy intake from protein was associated with an increased risk for the development of GADA, hazard ratio 1.24 (95% CI: 1.09, 1.53), *p*=0.042, which suggests that the findings from the mediation analysis showing an effect of energy from protein (kcal/day) on the development of GADA is not driven by the exclusion of participants who left the study before the age of 8 years. To further analyse this finding, we performed an additional Cox analysis replacing HLA with (1) HLA DR4 genotype (yes or no) and (2) HLA DR3 genotype (yes or no). For each of these analyses, we included the genotype and the interaction of the genotype with energy from protein. These analyses detected a significant interaction between energy from protein and HLA DR4 haplotype in relation to the development of GADA (*p*=0.017), but no significant interaction was found for energy from protein and HLA DR3 (*p*=0.761). The hazard ratio (95% CI) for energy from protein for DR4 no was 0.71 (0.43, 1.18) and for DR4 yes was 1.37 (1.10, 1.72).

## 4. Discussion

In this large multinational birth cohort of genetically predisposed children, we found that total energy intake (and energy from macronutrients, independently) from the age of two years, acts indirectly through BMI on the development of islet autoimmunity. Interestingly, there appears to be a direct effect of energy from protein on the development of GADA, where a larger contribution of energy from protein in the diet increases the risk of GADA, but not through increased BMI. When using energy adjusted macronutrient intake (energy percent), the indirect effect on development of islet autoimmunity disappeared but there was still a direct effect of percent of protein on the development of GADA, indicating that increasing amounts of protein appear to increase the risk of GADA, but not through increased BMI. In addition, it appears that the significant effect of protein on the development of GADA is primarily due to children who carry the DR4 haplotype.

This study confirms earlier data suggesting that higher BMI is associated with the development of islet autoimmunity and that excessive energy intake (greater energy intake than energy expenditure over a longer time) has an indirect effect on the outcome. Previous studies have used different measures of weight gain and obesity, such as rapid weight gain, length/height-for-age, BMI, and growth velocity, and most have shown an overall association between childhood overweight and obesity and subsequent increased risk of islet autoimmunity and type 1 diabetes [4, 19–21]. A plausible biological explanation is that increased weight gain or overweight increases insulin demands and influences beta-cell activity to indirectly modify the risk of developing islet autoimmunity. The novelty of this work is that it shows that energy from protein is contributing directly to the development of islet autoimmunity and specifically GADA positivity. It has been suggested that high protein diets during infancy and early childhood may accelerate growth and lead to overweight later in life. In two systemic reviews, it was found that higher protein intake during the second year of life was associated with increased risk of overweight and obesity in children [22, 23]. Also, studies have found that nondairy animal protein (meat) was associated with increased BMI; however, these findings have been inconsistent [24].

Higher protein intake increases the intake of essential branched-chain amino acids (BCAA), which has been associated with increased risk for overweight and insulin resistance in children [25]. High protein intake increases circulating concentrations of insulin-releasing BCAA, which stimulate the secretion of insulin and insulin-like growth factor (IGF-1) and consequently enhance weight gain [26]. Herein, we showed that energy intake from protein was associated with GADA positivity. To our knowledge, protein intake has not been related to islet autoimmunity or type 1 diabetes in any previous prospective study. Of note, a previous ecologic study showed that type 1 diabetes incidence in children was positively correlated with average per capita energy intake from food items of animal origin [27]. While it is difficult to draw conclusions from ecological studies, these observations further support the notion of associations among dietary habits, food intake patterns, and the incidence of childhood type 1 diabetes at population level. More recently, two studies found an association with higher meat consumption and increased risk of type 1 diabetes [28, 29]. Cereal consumption (plant protein) and high intake of cow's milk has been associated with islet autoimmunity and progression to type 1 diabetes in genetically susceptible children [30–33]. During early childhood, milk and milk products are often consumed in large amounts, especially in the Nordic countries, including Sweden and Finland. It has been reported that high milk intake, but not meat intake, increased the concentrations of IGF-1 and was associated with increased overweight and obesity later during childhood [34, 35].

The infant gut microbiome undergoes several phases of microbiome progression during the first four years of life where the major driver of changes during late infancy are breastfeeding duration and introduction to solid complementary foods [24, 36]. The dietary composition has a large effect on the microbiome where different dietary components directly shape the gut microbiota and diversity [37, 38]. A less healthy diet (high fat and/or protein and low carbohydrates) is often characterized by less favourable ratio between the two dominating phyla: Bacteroides to Firmicutes [39]. For example, following a Mediterranean diet, that includes high intakes of plant foods, unsaturated vegetable oils, dairy products, and low amounts of meat, which in turn leads to lower protein intake compared to a Western diet, has been inversely associated with not only the risk of overweight and obesity but also increased abundance of health-related bacterial species and short-chain fatty acids (SCFA) producing bacteria [40]. The human gut microbiota may play an important role in the development of obesity later in life but the exact mechanism by which the gut microbiota contribute to obesity is not well known and need to be investigated further.

In the present study, we do not have information about the food groups as a type of protein source (animal or plant) to conduct further in-depth analyses. These findings highlight the need for future studies on dietary patterns and food groups and their impact on the development of islet autoimmunity and progression to type 1 diabetes, especially during the transition period from milk feeding (either breastmilk or infant formula) to the long-term family eating habits. Previous research has highlighted that energy from protein should not exceed 15% in the child's diet during the first 12–24 months of life, with the aim to reduce the risk of obesity later in childhood [41, 42]. Daily protein intake in children living in high-income countries usually exceed by far the recommended intake. The dietary requirement, defined as minimum intake that meets the metabolic demands, maintenance of body protein mass, and the needs for growth in children, is 0.8–0.9 g protein/kg body weight, which equates to about 5 energy percent [43]. The corresponding protein intake in the current study ranges from 3.4 g/kg bodyweight at 2 years of age and 2.1 g/kg bodyweight at 8 years of age (data not shown). Moreover, the reported protein intake in this multinational study population aligns with reported intake from similar pediatric study populations [44, 45]. While more confirmatory data are needed, identifying modifiable risk factors for islet autoimmunity and type 1 diabetes in early life is needed. Studying the effects of different protein rich foods on growth during childhood or actively managing the proportion of macronutrients in pediatric diets could potentially provide a strategy for reducing risk for islet autoimmunity in genetically predisposed children.

Capturing a habitual food intake is difficult and study results may be affected by family dietary patterns and food choices due to parental knowledge of the child's genetic risk of type 1 diabetes. In TEDDY, we collect information about parental actions to prevent type 1 diabetes in the offspring. At the age of 15 months, 29% of the mothers reported dietary changes as an action to prevent type 1 diabetes in their child [46]. The specific type of dietary change most often reported was reducing the intake of carbohydrate rich foods in the child's diet (sweets or carbohydrates). The rationale behind maybe based on the belief that high intake of sugar rich foods will cause type 1 diabetes. These maternal actions may skew the ratio between the energy providing macronutrients.

Most studies have focused on rapid weight gain during the first 6–12 months of life but different growth phases have been identified, and rapid weight gain from two years of age and onwards has also been associated with the later risk of overweight and obesity [47]. The child's diet during this period is mostly influenced by family eating habits that maybe consistent throughout the childhood. Longer duration of breastfeeding (both exclusive and any) has been associated with slower weight gain during the first year of life [48] and findings from the TEDDY cohort indicate that longer breastfeeding duration (both exclusive and any) were associated with reduced risk of obesity at 5 years of age, but no association was found for the development of islet autoimmunity [49]. Despite this knowledge, it is of relevance to explore if dietary habits after breastfeeding period also are associated with growth, obesity, and the risk of islet autoimmunity during childhood.

### 4.1. Study Limitations

Due to difficulties in estimating total energy intake in breastfed children, a minimum age cut-off of two years was decided. In TEDDY, caregivers were instructed to only record each episode of breastfeeding in the 3-day food records. Daily energy intake in breastfed infants was calculated using an algorithm based on the child's energy requirements at a given age and body weight plus additional energy need for growth [50]. By using this method, it is difficult to capture potential under and overfeeding during infancy. The chosen minimum age cut-off of two years of age resulted in the exclusion of subjects who developed IA before the age of 2 years. The incidence rate of IAA as first islet autoantibody reaches a peak at one year of age, indicating a more rapid progression to the disease [18], and in this study population, children developed GADA at an older age compared to children who develop IAA. In addition, a total of 44 children developed type 1 diabetes before 2 years of age. The exclusion criteria may imply a better overview of the development of GADA compared to IAA. Of note, similar results have been described in the TEDDY study population before, showing a higher rate of weight gain during early childhood (up to the age of 4 years) was associated with an increased risk of progression from IA to type 1 diabetes in children with GADA as first appearing autoantibody [6].

An additional limitation is the inability of the software used for the mediation analyses to analyse censored observations. The larger categories of excluded subjects (withdrawn from the study before the age of 2 years, missing food record data and censored observations, i.e., left the study between the age of 2 and 8 years) could bias the observations of the present study. To address this, Cox proportional hazard analysis that included subjects who left the study before the age 8 was performed and showed a significant association of energy from protein and the development of GADA, supporting the findings from the mediation analysis.

Other factors associated with overweight and obesity later in life, such as maternal BMI, maternal type 1 diabetes status, birth weight, and breastfeeding duration have not been taken into consideration in the mediation analysis, and this limitation should be acknowledged when interpreting the results.

In conclusion, this study confirms that a higher total energy intake is associated with higher BMI, which in turn leads to the higher risk of development of islet autoimmunity. Further, by analysing dietary components, a diet with higher proportion of energy from protein has a direct effect on the development of GADA.

## Figures and Tables

**Table 1:** Descriptive characteristics for the study population consisting of islet autoantibody negative children in the TEDDY study (*n* = 5,084).

Baseline demographics	*N* (%)						
Country							
United States	1996 (39)						
Finland	1107 (22)						
Germany	243 (5)						
Sweden	1738 (34)						
HLA genotype							
DR3/DR4	1975 (39)						
DR4/DR4	1007 (20)						
DR4/DR8	846 (17)						
DR3/DR3	1076 (21)						
Other	180 (4)						
Female sex (yes)	2487 (49)						
First-degree relative with T1D (yes)	629 (12)						

Dietary intake and anthropometric data	2 years	3 years	4 years	5 years	6 years	7 years	8 years
	Median (IQR)						

Total energy intake (kcal/day)	1077 (938–1238)	1159 (1005–1338)	1235 (1065–1426)	1308 (1134–1512)	1384 (1185–1601)	1449 (1234–1679)	1501 (1271–1763)
Energy intake, protein (kcal/day)	169 (140–202)	179 (147–213)	188 (155–223)	198 (164–235)	208 (172–247)	217 (179–259)	225 (185–270)
Energy intake, fat (kcal/day)	349 (285–423)	370 (302–450)	399 (326–483)	425 (347–516)	451 (368–547)	475 (384–579)	501 (404–615)
Energy intake, carbohydrates (kcal/day)	546 (469–643)	599 (508–703)	637 (542–746)	675 (569–796)	709 (593–833)	741 (617–874)	758 (629–908)
Percentage of energy from protein (%)	15.7 (13.9–17.6)	15.4 (13.5–17.4)	15.2 (13.4–17.1)	15.1 (13.2–17.1)	15.1 (13.2–17.1)	15.1 (13.2–17.1)	15.1 (13.3–17.1)
Percent of energy from fat (%)	32.6 (28.5–36.8)	32.2 (28.4–35.9)	32.4 (28.6–36.3)	32.6 (28.9–36.6)	32.9 (29.1–36.6)	33.0 (29.4–36.7)	33.5 (29.8–37.4)
Percent of energy from carbohydrates (%)	51.3 (46.9–55.7)	52.2 (47.9–56.3)	52.1 (47.8–56.2)	51.8 (47.6–56.0)	51.7 (47.5–55.7)	51.5 (47.3–55.6)	51.1 (46.8–55.2)
BMI (*z*-score)	0.15 (−0.53–0.77)	0.27 (−0.42–0.91)	0.33 (−0.36–0.96)	0.32 (−0.36–0.94)	0.27 (−0.36–0.90)	0.23 (−0.41–0.88)	0.21 (−0.44–0.89)

**Table 2:** Estimates (95% confidence interval) for total effect, direct effect, and indirect effect for islet autoantibody development (outcome) in TEDDY children (*n* = 5,084), using BMI *z*-score as the mediator in all analyses.

Exposure variable	Outcome	Total effect	Direct effect	Indirect effect
		Estimates (95% CI)		
Total energy intake (kcal)	IA positivity (≥1)	0.89 (−1.43, 2.20)	0.76 (−1.63, 2.20)	**0.13 (0.05, 0.21)**
	IAA	−0.25 (−2.47, 1.87)	−0.39 (−2.49, 1.84)	**0.14 (0.00, 0.20)**
	GADA	1.15 (−0.48, 2.28)	1.03 (−0.55, 2.22)	0.12 (−0.01, 0.26)

Energy intake from protein (kcal)	IA positivity (≥1)	0.83 (−0.41, 1.45)	0.78 (−0.47, 1.41)	**0.06 (0.02, 0.11)**
	IAA	−0.09 (−1.31, 0.91)	−0.08 (−1.31, 0.87)	0.04 (−0.03, 0.12)
	GADA	**1.14 (0.36, 1.56)**	**1.09 (0.35, 1.56)**	0.06 (−0.03, 0.14)

Energy intake from fat (kcal)	IA positivity (≥1)	0.22 (−0.32, 0.54)	0.19 (−0.35, 0.53)	**0.03 (0.01, 0.05)**
	IAA	−0.06 (−0.61, 0.38)	−0.10 (−0.64, 0.38)	0.05 (−0.04, 0.08)
	GADA	0.27 (−0.21, 0.54)	0.26 (−0.29, 0.56)	0.01 (−0.05, 0.09)

Energy intake from carbohydrates (kcal)	IA positivity (≥1)	0.06 (−0.41, 0.29)	0.04 (−0.44, 0.28)	**0.02 (0.00, 0.04)**
	IAA	−0.07 (−0.48, 0.24)	−0.06 (−0.51, 0.25)	0.03 (−0.03, 0.16)
	GADA	−0.01 (−0.30, 0.22)	−0.02 (−0.35, 0.24)	0.01 (−0.05, 0.09)

The bold values represent the 95% confidence intervals not going through 0 which are considered as significant effect.

**Table 3:** Estimates (95% CI) for total effect, direct effect, and indirect effect for islet autoantibody development (outcome), using energy percentage (energy from macronutrients/total energy) as exposure variables among TEDDY children (*n* = 5,084). In all analyses, BMI *z*-score is used as mediator.

Exposure variable	Outcome	Total effect	Direct effect	Indirect effect
		Estimates (95% CI)	Estimates (95% CI)	Estimates (95% CI)
Percent of energy from protein (%)	IA positivity (≥1)	40.6 (−7.8, 97.8)	39.6 (−7.9, 97.8)	1.0 (−0.2, 2.4)
	IAA	33.0 (−4.7, 98.1)	31.7 (−4.7, 98.1)	1.4 (−0.2, 2.6)
	GADA	**73.7 (3.9, 98.0)**	**72.8 (3.0, 98.0)**	0.9 (−1.3, 3.3)

Percent of energy from fat (%)	IA positivity (≥1)	3.2 (−9.4, 17.2)	3.0 (−9.3, 16.9)	0.2 (−0.2, 0.9)
	IAA	0.2 (−6.4, 15.5)	0.1 (−0.1, 0.6)	0.1 (−0.1, 0.6)
	GADA	4.3 (−8.8, 50.8)	4.2 (−8.9, 50.3)	0.2 (−0.2, 0.9)

Percent of energy from carbohydrates (%)	IA positivity (≥1)	−5.8 (−20.3, 7.2)	−5.7 (−20.2, 7.1)	−0.1 (−0.4, 0.0)
	IAA	−2.0 (−16.7, 4.7)	−2.0 (−16.7, 4.8)	−0.1 (−0.4, 0.0)
	GADA	−8.5 (−24.8, 5.5)	−8.4 (−24.8, 6.0)	−0.1 (−0.5, 0.1)

The bold values represent the 95% confidence intervals not going through 0 which are considered as significant effect.

## Data Availability

The datasets generated and analysed during the current study are available in the NIDDK Central Repository at https://repository.niddk.nih.gov/studies/teddy.

## Appendices

### A. TEDDY Study Group

Colorado Clinical Center: Marian Rewers, M.D., Ph.D., PI, Aaron Barbour, Kimberly Bautista, Judith Baxter, Daniel Felipe-Morales, Brigitte I. Frohnert, M.D., Marisa Stahl, M.D., Patricia Gesualdo, Michelle Hoffman, Rachel Karban, Edwin Liu, M.D., Alondra Munoz, Jill Norris, Ph.D., Holly O'Donnell, Ph.D., Stesha Peacock, Hanan Shorrosh, Andrea Steck, M.D., Megan Stern,Kathleen Waugh. University of Colorado, Anschutz Medical Campus, Barbara Davis Center for Childhood Diabetes.

Finland Clinical Center: Jorma Toppari, M.D., Ph.D., PI¥^, Olli G. Simell, M.D., Ph.D., Annika Adamsson, Ph.D.^, Sanna-Mari Aaltonen^, Suvi Ahonen*∗*±§, Mari Åkerlund*∗*±§, Leena Hakola*∗*±, Anne Hekkala, M.D. *μ*¤, Henna Holappa *μ*¤, Heikki Hyöty, M.D., Ph.D.*∗*±, Anni Ikonen *μ*¤, Jorma Ilonen, M.D., Ph.D. ¥, SAnna Jokipuu^, Leena Karlsson^, Jukka Kero M.D., Ph.D. ¥^, Jaakko J. Koskenniemi M.D., Ph.D.¥^, Miia Kähönen *μ*¤, Mikael Knip, M.D., Ph.D.*∗*±, Minna-Liisa Koivikko *μ*¤, Katja Kokkonen*∗*±, Merja Koskinen*∗*±, Mirva Koreasalo*∗*±§, Kalle Kurppa, M.D., Ph.D. *∗*±, Salla Kuusela, M.D. *∗*±, Jarita Kytölä *∗*±, Jutta Laiho, Ph.D. *∗*, Tiina Latva-aho *μ*¤, Laura Leppänen^, Katri Lindfors, Ph.D.*∗*, Maria Lönnrot, M.D., Ph.D.*∗*±, Elina Mäntymäki^, Markus Mattila*∗*±, Maija Miettinen§, Katja Multasuo *μ*¤, Teija Mykkänen *μ*¤, Tiina Niininen±*∗*, Sari Niinistö§, Mia Nyblom*∗*±, Sami Oikarinen, Ph.D.*∗*±, Paula Ollikainen *μ*¤, Zhian Othmani¥, Sirpa Pohjola *μ*¤, Jenna Rautanen±§, Anne Riikonen*∗*±§, Minna Romo^, Satu Simell, M.D., Ph.D.¥, Päivi Tossavainen, M.D.*μ*¤, Mari Vähä-Mäkilä¥, Eeva Varjonen^, Riitta Veijola, M.D., Ph.D.*μ*¤, and Irene Viinikangas *μ*¤, Suvi M. Virtanen, M.D., Ph.D.*∗*±§. ¥University of Turku, *∗*Tampere University, *μ*University of Oulu, ^Turku University Hospital, Hospital District of Southwest Finland, ±Tampere University Hospital, ¤Oulu University Hospital, §Finnish Institute for Health and Welfare, Finland, University of Kuopio.

Georgia/Florida Clinical Center: Jin-Xiong She, Ph.D., PI, Desmond Schatz, M.D.*∗*, Diane Hopkins, Leigh Steed, Jennifer Bryant, Katherine Silvis, Michael Haller, M.D.*∗*, Melissa Gardiner, Richard McIndoe, Ph.D., Ashok Sharma, Stephen W. Anderson, M.D.^, Laura Jacobsen, M.D.*∗*, and John Marks, DHSc., P.D. Towe*∗*. Center for Biotechnology and Genomic Medicine, Augusta University. *∗*University of Florida, Pediatric Endocrinology. ^Pediatric Endocrine Associates, Atlanta.

Germany Clinical Center: Anette G. Ziegler, M.D., PI, Ezio Bonifacio Ph.D.∗, Cigdem Gezginci, Anja Heublein, Eva Hohoff¥, Sandra Hummel, Ph.D., Annette Knopff, Charlotte Koch, Sibylle Koletzko, M.D., Claudia Ramminger, Roswith Roth, Ph.D., Jennifer Schmidt, Marlon Scholz, Joanna Stock, Katharina Warncke, M.D., and Lorena Wendel, Christiane Winkler, Ph.D. Forschergruppe Diabetes e.V. and Institute of Diabetes Research, Helmholtz Zentrum München, Forschergruppe Diabetes, and Klinikum rechts der Isar, Technische Universität München. ∗Center for Regenerative Therapies, TU Dresden, Dr. von Hauner Children's Hospital, Department of Gastroenterology, Ludwig Maximillians University Munich, ¥University of Bonn, Department of Nutritional Epidemiology.

Sweden Clinical Center: Åke Lernmark, Ph.D., PI, Daniel Agardh, M.D., Ph.D., Carin Andrén Aronsson, Ph.D., Rasmus Bennet, Susanne Dahlberg, Ulla Fält, Malin Goldman Tsubarah, Emelie Ericson-Hallström, Lina Fransson, Thomas Gard, Emina Halilovic, Gunilla Holmén, Susanne Hyberg, Berglind Jonsdottir, M.D., Ph.D., Naghmeh Karimi, Helena Elding Larsson, M.D., Ph.D., Marielle Lindström, Markus Lundgren, M.D., Ph.D., Marlena Maziarz, Ph.D., Maria Månsson Martinez, Jessica Melin, Zeliha Mestan, Caroline Nilsson, Yohanna Nordh, Kobra Rahmati, Anita Ramelius, Falastin Salami, Anette Sjöberg, Carina Törn, Ph.D., Ulrika Ulvenhag, Terese Wiktorsson, and Åsa Wimar. Lund University.

Washington Clinical Center: William et al., Washington Clinical Center: William A. Hagopian, M.D., Ph.D., PI, Michael Killian, Claire Cowen Crouch, Jennifer Skidmore, Luka-Sophia Bowen, Mikeil Metcalf, Arlene Meyer, Jocelyn Meyer, Denise Mulenga, Nole Powell, Jared Radtke, Shreya Roy, Davey Schmitt, Preston Tucker. Pacific Northwest Research Institute.

Pennsylvania Satellite Center et al., Pennsylvania Satellite Center: Dorothy Becker, M.D., Margaret Franciscus, MaryEllen Dalmagro-Elias Smith, Ashi Daftary, M.D., Mary Beth Klein, Chrystal Yates. Children's Hospital of Pittsburgh of UPMC.

Data Coordinating Center: Jeffrey P. Krischer, Ph.D., PI, Rajesh Adusumali, Sarah Austin-Gonzalez, Maryouri Avendano, Sandra Baethke, Brant Burkhardt, Ph.D., Martha Butterworth, Nicholas Cadigan, Joanna Clasen, Kevin Counts, Laura Gandolfo, Jennifer Garmeson, Veena Gowda, Christina Karges, Shu Liu, Xiang Liu, Ph.D., Kristian Lynch, Ph.D., Jamie Malloy, Lazarus Mramba, Ph.D., Cristina McCarthy, Jose Moreno, Hemang M. Parikh, Ph.D., Cassandra Remedios, Chris Shaffer, Susan Smith, Noah Sulman, Ph.D., and Roy Tamura, Ph.D. roy.tamura@epi.usf.edu, Dena Tewey, Michael Toth, Ulla Uusitalo, Ph.D. ulla.uusitalo@epi.usf.edu, Kendra Vehik, Ph.D. kendra.vehik@epi.usf.edu, Ponni Vijayakandipan, Melissa Wroble, Jimin Yang, Ph.D. jimin.yang@epi.usf.edu, R. D., and Kenneth Young, Ph.D. Past staff: Michael Abbondondolo, Lori Ballard, Rasheedah Brown, David Cuthbertson, Stephen Dankyi, Christopher Eberhard, Steven Fiske, David Hadley, Ph.D., Kathleen Heyman, Belinda Hsiao, Francisco Perez Laras, Hye-Seung Lee, Ph.D., Qian Li, Ph.D., Colleen Maguire, Wendy McLeod, Aubrie Merrell, Steven Meulemans, Ryan Quigley, and Laura Smith, Ph.D. University of South Florida.

Project scientist: Beena Akolkar, Ph.D. National Institutes of Diabetes and Digestive and Kidney Diseases.

Autoantibody Reference Laboratories: Liping Yu, M.D.^, Dongmei Miao, M.D.^, Kathleen Gillespie∗, Kyla Chandler∗, Ilana Kelland∗, Yassin Ben Khoud∗, Matthew Randell∗. ^Barbara Davis Center for Childhood Diabetes, University of Colorado Denver, ∗Bristol Medical School, University of Bristol, UK.

HLA Reference Laboratory: William Hagopian, M.D., Ph.D., Jared Radtke, Preston Tucker. Pacific Northwest Research Institute, Seattle, WA, USA. (Previously Henry Erlich, Ph.D., Steven J. Mack, Ph.D., and Anna Lisa Fear. Center for Genetics, Children's Hospital Oakland Research Institute.)

Repository: Sandra Ke, Niveen Mulholland, Ph.D. NIDDK Biosample Repository at Fisher BioServices, Rockville, MD, USA.

Other contributors: Thomas Briese, Ph.D., Columbia University. Todd Brusko, Ph.D., University of Florida, Gainesville, FL, USA. Suzanne Bennett Johnson, Ph.D., Florida State University, Tallahassee, FL, USA. Eoin McKinney, Ph.D., University of Cambridge, Cambridge, UK. Tomi Pastinen, M.D., Ph.D., The Children's Mercy Hospital, Kansas City, MO, USA. Steffen Ullitz Thorsen, M.D., Ph.D., Department of Clinical Immunology, University of Copenhagen, Copenhagen, Denmark, and Department of Pediatrics and Adolescents, Copenhagen University Hospital, Herlev, Denmark. Eric Triplett, Ph.D., University of Florida, Gainesville, FL, USA.

## Supplementary Materials

Supplementary Materials Figure S1 describes the time-varying mediation analysis and Table S1 shows a cross-tabulation of BMI *z*-score vs. islet autoimmunity at the ages 3 through 8.
