# Supplementary material for: Dietary Intake and Body Mass Index Influence the Risk of Islet Autoimmunity in Genetically At-Risk Children: A Mediation Analysis Using the TEDDY Cohort
Source: Pediatr Diabetes. 2023 Feb 17;2023:3945064. doi: 10.1155/2023/3945064 (PMC10445692; doi:10.1155/2023/3945064)
Supplement: Supplementary Materials — Figure S1 describes the time-varying mediation analysis and Table S1 shows a cross-tabulation of BMI z-score vs. islet autoimmunity at the ages 3 through 8. [file 3945064.f1.pdf]

Supplementary material for Andrén Aronsson et al. ***Dietary intake and body mass index influences the risk of islet autoimmunity in genetically at-risk children: a mediation analysis using the TEDDY cohort.***

Figure S1. Time-varying mediation with ordering of variables of A(t), M(t), L(t). Causal graphic where (L) represents confounders, (A) represents exposure, (M) represents mediator, and (Y) is the final outcome at a fixed time point. Note that Y replaces L(t) at the final time point. Abbreviations; L(0) = baseline covariates; HLA-genotype, country, and first degree relative with T1D. M(1) = growth, BMI z-scores at time point i.A(1) = energy intake from time point i-1. Figure by the authors WanderVeele and Tchetgen. (17), reprinted with permission from the Royal Statistical Society and © 2021 John Wiley & Sons, Inc.

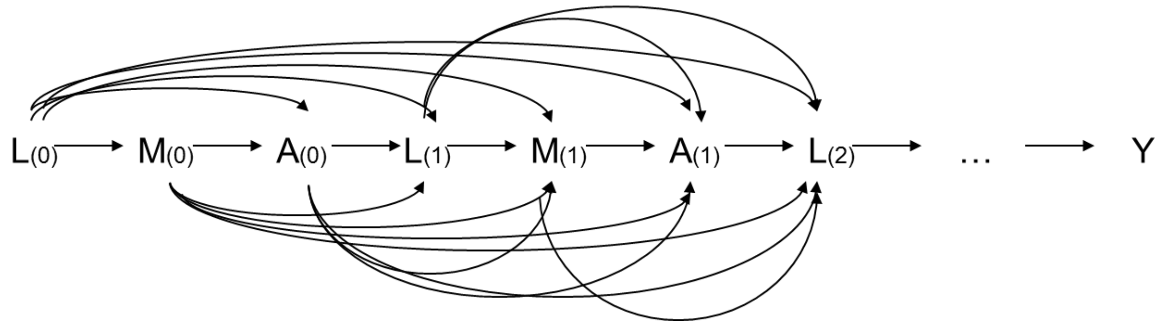

Table S1. Cross tabulation of BMI z-score ≥1.5 versus IA positivity at ages 3 through 8.

	Age											
	**3**		**4**		**5**		**6**		**7**		**8**	
	IA + (%)	N	IA + (%)	N	IA + (%)	N	IA + (%)	N	IA + (%)	N	IA + (%)	N
BMI (z-score) <1.5	92 (2.0)	4502	165 (3.7)	4453	215 (4.9)	4433	253 (5.7)	4462	285 (6.4)	4464	318 (7.2)	4418
BMI (z-score) ≥1.5	15 (3.4)	447	21 (4.2)	496	23 (4.5)	516	31 (6.4)	487	36 (7.4)	485	42 (7.9)	531
